# Tidal volume according to the 4-point sealing forces of a bag-valve-mask: an adult respiratory arrest simulator-based prospective, descriptive study

**DOI:** 10.1186/s12873-021-00451-1

**Published:** 2021-05-01

**Authors:** Dongchoon Uhm, Ajung Kim

**Affiliations:** 1 Department of Emergency Medical Technology, Daejeon University, 62 Daehak-ro, Dong-gu, Daejeon, 34520 Republic of Korea; 2 Department of Emergency Medical Technology, Kyungil University, 50, Gamasil-gil, Hayang-eup, Gyeongsan-si, Gyeongbuk 38428 Republic of Korea

**Keywords:** Tidal volume, Bag-valve-mask, Ventilation

## Abstract

**Background:**

For adequate ventilation during bag-valve-mask ventilation, rescuers should ensure a proper mask seal using the one-handed or two-handed technique. Little is known about the magnitude of sealing forces of a bag-valve-mask needed for adequate ventilation. This study aimed to explore the effect of the 4-point sealing forces of a bag-valve-mask on tidal volume while using the one-handed technique, focusing on the moderating effect of C length (the distance from the thumb to the index finger in the C shape of the one-hand EC grip).

**Methods:**

A prospective, descriptive simulation study was conducted. A convenience sample of 125 undergraduate paramedic students from two universities was recruited. A self-reported questionnaire was used to collect subjective variables. Tidal volumes, 4-point sealing forces of the mask, peak pressure, and C length of the C shape in the one-hand EC grip were measured using the mechanical lung model under a simulated adult respiratory arrest. Hierarchical regression analysis was used to determine the moderating effect of C length on tidal volume in bag-valve-mask ventilation.

**Results:**

The average C length, peak pressure, and tidal volume were 7.54 (± 1.85) cm, 11.62 (± 5.40) cmH_2_O, and 321.66 (± 135.18) mL, respectively. The average range of the 4-point sealing forces was 0.03–0.69 N. The apex sealing force was the weakest among the 4-point sealing forces. Hierarchical regression analysis demonstrated that tidal volume accounted for 62.7% of the variance by C length, peak pressure, and apex sealing force during bag-valve-mask ventilation (F = 9.676, *p* < 0.001). C length moderated the effect of the apex sealing force and peak pressure on the tidal volume, meaning the higher the peak pressure and apex sealing force, the more the tidal volume and the longer the C length.

**Conclusion:**

This first simulation study measuring the 4-point sealing forces during bag-valve-mask ventilation provides effective advice that can be adopted in clinical practice without side effects and underpins the importance of continuous retraining and assessment focused on individual physical characteristics, such as C length and bag-valve-mask sealing forces.

## Background

Bag-valve-mask (BVM) ventilation is the standard treatment for cardiopulmonary arrest, as well as emergency airway management in cases where spontaneous breathing is failing or has ceased, regardless of the place or time. The delivery of adequate BVM ventilation by rescuers is considered relatively simple; however, it is an important and challenging skill that requires considerable practice. Adequate ventilation involves delivering each rescue breathing over 1 s to provide a sufficient tidal volume (TV), using the BVM, and produce a visible chest rise [1]. This is deeply associated with the survival rate as well as oxygenation of patients with cardiac or respiratory arrest [2]. However, excessive ventilation results in significantly increased intrathoracic pressure and decreased coronary perfusion pressures and survival rates [3, 4]. Rescuers seal the mask against the face using either one- or two-handed techniques, with the thumb and the index finger wrapped in a “C” shape around the mask apex, and the remaining fingers in an “E” shape, lifting the jaw [5]; this is called the “EC grip”. Successful ventilation via the EC grip can be achieved by creating a tight seal between the mask and face and squeezing the bag with reasonable force [6]. Several previous studies [7–17] have reported that the TV delivered during BVM ventilation is affected by rescuers’ squeezing methods, such as the thenar eminence technique, E-O technique, and E-C technique, as well as individual characteristics, such as gender, height, weight, hand width and length, grip power, education, practice, and experience, among others; however, the reported results have been inconsistent.

The most important aspect of air leaking prevention during BVM ventilation is the perfect seal between the mask and patient’s face, generated by the sealing force of the mask toward the patient’s face during the EC grip. A few studies have explored the relationship between BVM ventilation and sealing force in South Korea; therefore, the purpose of this study was to identify the factors affecting the TVs with respect to the 4-point sealing forces of BVMs, focusing on the moderating effects of C length, using a RespiTrainer® Advance manikin and Quick Lung®.

## Material and methods

### Study design

This was a prospective, descriptive study that simulated adult respiratory arrest using a mechanical lung model.

### Participants and setting

To ensure an adequate sample size, we performed a power analysis using the G Power 3.1.5 (SOFTMEDIA) program; the desired sample size for computing a test power (1-β) of 0.80 with 17 predictors was 146, with an effect size of 0.15 and alpha of 0.05. After contacting the directors of two universities from a big city in South Korea to obtain permission for recruitment, a convenience sample of 160 undergraduate paramedic students from two universities was recruited; 35 students dropped out due to personal reasons, such as part-time jobs or family events. Finally, 125 questionnaires were used in the analysis; participation was both voluntary and anonymous. The inclusion criterion was right-handed individuals who had completed the cardiopulmonary resuscitation (CPR) theory and practical course according to the 2015 American Heart Association (AHA) guidelines.

### Measurements and procedures

Measurements in this study consisted of both subjective and objective variables. Subjective variables were measured using the self-reported questionnaire, which included general characteristics, knowledge, and confidence in performing the BVM ventilation technique for respiratory arrest patients; this took approximately 10 min to complete. Data collection of objective variables, such as TV, peak pressure (PP), C length, and 4-point sealing forces of the mask, took approximately 5 min per participant.

### Subjective variables

#### General characteristics

General characteristics of interest included gender, school year, academic grade, clinical practice satisfaction, major satisfaction, experience in the BVM ventilation of respiratory arrest patients, acquired certificates, and body mass index (BMI, kg/m^2^).

#### Knowledge

Knowledge regarding BVM ventilation was assessed using a previously published scale [14]. The questionnaire comprised 10 items; a correct response was scored 1. The total score ranged from 0 to 10, with high scores indicating more knowledge regarding BVM ventilation. The Kuder-Richardson Formula 20 was 0.69 in a previous study [14], and 0.68 in the present study.

#### Performance confidence

The scale used to assess performance confidence was developed in a previous study [14]. The questionnaire comprises ten items, with each rated on a five-point Likert scale ranging from “strongly disagree” [1], to “strongly agree” [5]. High scores indicated a higher level of performance confidence. Cronbach’s alpha in the previous study [14] was 0.90, and 0.93 in the present study.

#### Objective variables

The objective variables in this study included TV, PP, C length, and 4-point sealing forces of the mask. All participants were asked to hold the mask with their left hand and the bag with their right hand; data collection took approximately 5 min per participant.

### Experimental environment

#### TV and PP

A RespiTrainer® Advance manikin (IngMar Medical, Ltd., Pittsburgh, PA, USA) was placed at the same height as the participants’ middle femur line [17, 18], using the foothold to adjust the height. A Quick Lung® (IngMar Medical) and personal digital assistant were connected to the manikin, and data were sent to the personal digital assistant through its embedded device. The Quick Lung® was set with compliance of 50 mL·cmH_2_O^− 1^ and resistance of 5 cmH_2_O·L^− 1^·s based on previous studies [19, 20]. Participants were instructed to use a BVM (1600 mL, Ambu Mark IV- Reusable Resuscitator with silicon face mask size 5; Ambu, Copenhagen, Denmark) to ventilate the manikin, simulating an adult with respiratory arrest, for 2 min (measured using a metronome (10 times/min)). BVM ventilation was performed while standing without chest compression and oral or nasal airway; i.e., the left hand secured the mask on the manikin’s face using the “EC” grip, while the right hand held the bag comfortably. Before data collection, participants were given the opportunity to practice ventilation for 2 min to familiarize themselves with the study setting. The study participants were blinded to the study purpose, and the TV (mL) and PP (cmH2O) were collected for each participant and the averages were calculated for each of them.

#### Apex, bottom, left, and right sealing forces of the mask in C shape of EC grip

**Fig. 1:**
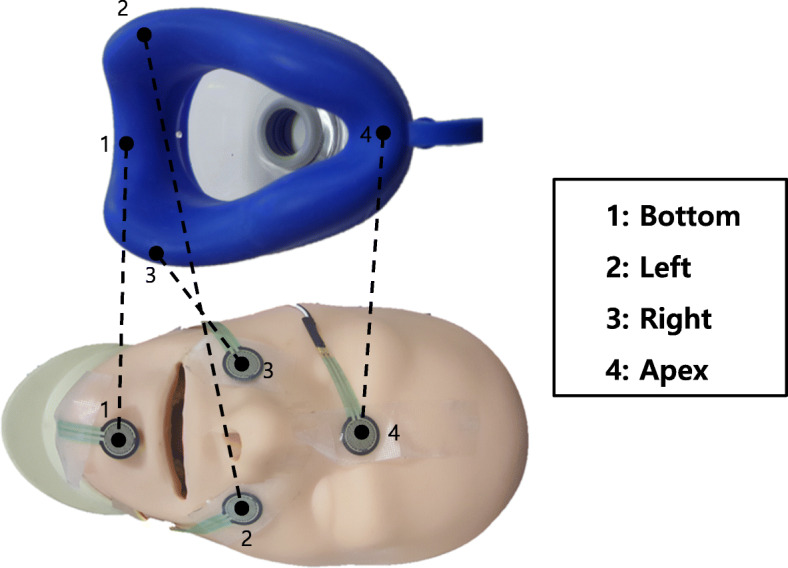
1: The area where the chin and the bottom of the mask adhere. 2: The area where the left side of the face and the left edge of the mask come into close contact. 3: The area where the right side of the face and the right tip of the mask are in close contact. 4: The area where the nose and the apex tip of the mask are in close contact

The 4-point forces of the mask in the EC grip are the apex, bottom, left, and right sealing forces. To measure the sealing forces in the C shape of the EC grip, sensors were attached to four points where the mask touched the manikin’s face (Fig. 1). Four 0.5″ Circles (Part No. 402) of Interlink Force.

Sensing Registors® (Interlink Electronics, Inc., Camarillo, California, United States) were installed for force measurement; the force data (N) collected from the sensor were coded and calculated through ARDUINO Software [21].

#### C length

**Fig. 2:**
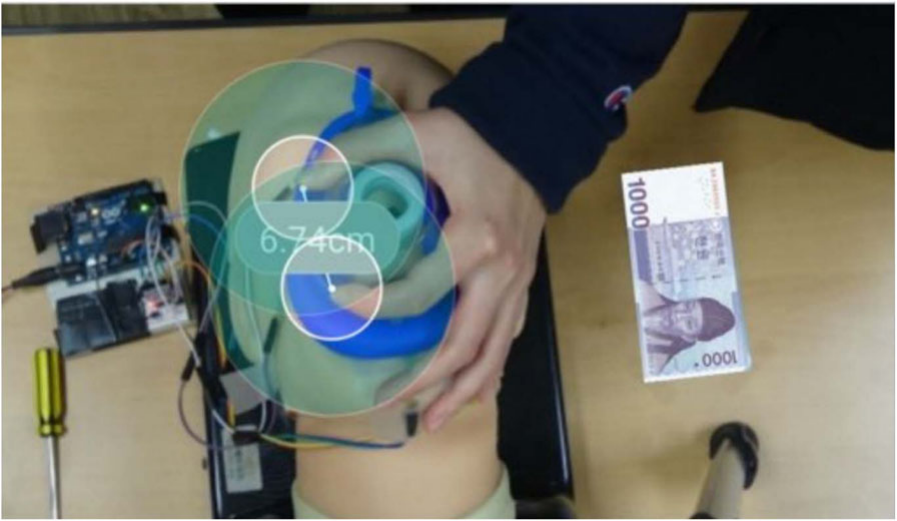
The length was measured after the start and end points of the reference object were fitted to the thumb and index fingers in the picture(The reference object for this study was a 1,000 won Korean bill)

The C length (cm), defined as the distance from the thumb to the index finger in the C shape of the “EC” grip, was measured using a distance-measuring application from AGUMON LAB [22]. First, a picture of the EC-shaped hand that held the BVM was taken. Subsequently, a reference object was selected for length measurement (the reference object for this study was a 1000 won Korean bill; Fig. 2), and the start and end points of the reference object were similarly selected. Finally, the length was measured after the start and end points of the reference object were fitted to the thumb and index fingers in the picture.

### Statistical analysis

Data were analyzed using IBM SPSS Statistics for Windows, Version 26.0 (SPSS Inc., Chicago, IL, USA). To confirm normal distribution of the data, the kurtosis and skewness were confirmed. Logarithmic and squared transformation was applied on apex sealing force data; the kurtosis and skewness were less than the standard of normality (skewness < 3, kurtosis < 10). Statistical analyses were performed via descriptive statistics, Pearson’s correlation analysis, and hierarchical regression analysis. To verify the moderating effects of C length on tidal volumes, general characteristics and subjective variables (knowledge, performance confidence) were adjusted (controlled), and hierarchical regression analysis was performed to confirm the influence of the predictors. Mean centering was used to remove multicollinearity among predictor variables (PP, apex sealing force, bottom sealing force, right sealing force, and left sealing force) and interaction variables (PP * C length; apex sealing force * C length; bottom sealing force * C length; right sealing force * C length; left sealing force * C length). Additionally, to resolve the problem of multicollinearity, the independent and moderating variables were standardized. Control variables, including gender, school year, academic grade, experience with BVM ventilation, and acquired certificate, were transformed into dummy codes; continuous variables, such as clinical practice satisfaction, major satisfaction, BMI, knowledge, and confidence performing BVM ventilation, were used without transformation.

### Ethical considerations and data collection

This study was conducted in accordance with the principles of the Declaration of Helsinki and was approved by the relevant Institutional Review Board. Written informed consent was obtained from all participants prior to conducting this study; the participants were informed that they could withdraw their consent at any point during this study without consequences*.* Data were collected between November 4, 2019, and December 13, 2019. Additionally, to prevent the intervention of the researchers, the research assistant, an emergency medical technician, was trained to collect data. In addition, a unique number was assigned to protect the personal information of the participants.

## Results

### General characteristics

General characteristics are presented Table 1. The average C length and PP were 7.54 (± 1.85) cm and 11.62 (± 5.40) cmH_2_O, respectively. The 4-point sealing forces (apex, bottom, left, and right) in the C shape of the EC grip were 0.03 (± 0.03) N, 0.69 (± 0.80) N, 0.53 (± 0.46) N, and 0.31 (± 0.41) N, respectively; the mean TV was 321.66 (± 135.18) mL.

**Table 1:** General characteristics and subjective and objective variables *N* = 125

Category				N	(%)	Mean (±SD)
General characteristics	Gender	male		58	(46.4)	
		female		67	(53.6)	
	School year	Freshman		31	(24.8)	
		Sophomore		30	(24.0)	
		Junior		54	(43.2)	
		Senior		10	(8.0)	
	Academic grades	4.0≤		20	(16.0)	
		3.0–3.99		84	(67.2)	
		2.99≥		21	(16.8)	
	Clinical practice satisfaction (1–5)					3.76 (±0.78)
	Major satisfaction (1–5)					4.07 (±0.76)
	Experience of BVM ventilation	No		69	(55.2)	
		Yes		56	(44.8)	
	Acquired certificate	No	0	59	(47.2)	
		Yes	1	52	(41.6)	
			2	9	(7.2)	
			3	5	(4.0)	
	BMI (kg/m^2^)					22.89 (±2.77)
Subjective variables	Knowledge (0–10)					6.29 (±1.69)
	Performance confidence (10–50)					36.89 (±6.80)
Objective variables	C length (cm)					7.54 (±1.85)
	Peak pressure (cmH_2_O)					11.62 (±5.40)
	ln (apex sealing force) (N)					0.03 (±0.03)
	Bottom sealing force (N)					0.69 (±0.80)
	Left sealing force (N)					0.53 (±0.46)
	Right sealing force (N)					0.31 (±0.41)
	Tidal volume (mL)					321.66 (±135.18)

ln (apex sealing force) = ln (apex sealing force + 1)^8^

### Correlation among major variables

The correlation among major variables is presented in Table 2. TV had positive correlations with PP (*r* = 0.744, *p* < 0.001), right sealing force (*r* = 0.409, *p* < 0.001), and acquired certificate (*r* = 0.299, *p* = 0.001). In addition, apex sealing force (*r* = 0.246, *p* = 0.06), experience in BVM ventilation (*r* = 0.230, *p* = 0.010), and knowledge (*r* = 0.205, *p* = 0.022), were correlated. However, TV had a negative correlation with C length (*r* = − 0.223, *p =* 0.012).

**Table 2:** Correlation among major variables

Category	Variables	*R*	(*p*)
Objective variables	Tidal volume	**1**	
	C length	−0.223^*^	*(0.012)*
	Peak Pressure (PP)	0.744^***^	*(< 0.001)*
	ln (apex sealing force)	0.246^**^	*(0.006)*
	Bottom sealing force	*−0.165*	*(0.065)*
	Left sealing force	*−0.068*	*(0.449)*
	Right sealing force	.409^***^	*(< 0.001)*
General characteristics	Clinical practice satisfaction	*−0.054*	*(0.548)*
	Major satisfaction	*0.039*	*(0.666)*
	Experience of BVM ventilation	0.230^*^	*(0.010)*
	Acquired certificate	0.299^*^	*(0.001)*
	BMI (kg/m^2^)	*0.100*	*(0.269)*
Subjective variables	Knowledge	0.205^*^	*(0.022)*
	Performance confidence	*0.144*	*(0.110)*

ln (Apex sealing force) = ln (Apex sealing force + 1)^8^, ^*^p < 0.05, ^**^p < 0.01, ^***^p < 0.001

### The moderating effects of C length in relation to TVs and 4-point sealing forces of the BVM

The results of the regression analysis on the moderating effects of C length are presented in Table 3. In the first step, hierarchical regression analysis was performed with general characteristics, subjective variables, PP, and 4-point sealing forces as independent variables to identify the effects of predictor variables on TV. PP and apex sealing force were significantly associated with the TV for Model 1, accounting for 59.7% of the explained variance in TV (F = 11.210, *p* < 0.001). Second, C length was added to Model 1; PP and apex sealing force were significantly associated with the TV, resulting in 59.6% of the explained variance in TV (F = 10.614, *p* < 0.001). Finally, interaction variables were added to Model 2 to identify the moderating effects of C length; among the interaction variables, PP _*_ C length (β = 0.195, *p* = 0.017) and apex sealing force _*_ C length (β = 0.154, *p* = 0.014) were significantly associated with TV. Model 3’s equation resulted in 62.7% of the explained variance in TV (F = 9.676, *p* < 0.001); i.e., C length was the variable found to have a moderating effect in the relationship between TV, PP, and apex sealing force.

**Table 3:** Moderating effects of C length

	MODEL I					MODEL II					MODEL III				
	B	S.E.	*β*	t	*p*	B	S.E.	*β*	t	*p*	B	S.E.	*β*	t	*p*
(Constant)	310.872	47.135		6.595^***^	< 0.001	307.808	47.382		6.496^***^	< 0.001	334.357	47.89		6.982^***^	< 0.001
PP	100.495	10.254	0.743	9.800^***^	< 0.001	99.688	10.324	0.737	9.656^***^	< 0.001	94.404	10.149	0.698	9.302^***^	< 0.001
ln (ASF)	17.521	8.789	0.13	1.993^*^	0.049	18.055	8.832	0.134	2.044^*^	0.043	14.649	8.554	0.108	1.712	0.09
BSF	6.5	10.192	0.048	0.638	0.525	6.342	10.212	0.047	0.621	0.536	4.327	9.856	0.032	0.439	0.662
LSF	4.409	9.854	0.033	0.447	0.655	3.907	9.892	0.029	0.395	0.694	2.139	9.583	0.016	0.223	0.824
RSF	−3.477	10.918	−0.026	−0.318	0.751	−4.074	10.965	−0.03	−0.372	0.711	−3.596	11.616	−0.027	− 0.31	0.758
Gender															
male	11.739	18.759	−0.043	− 0.626	0.533	13.801	18.976	0.051	−0.727	0.469	2 < .001	18.86	0.007	−0.106	0.916
School year															
first	2.92	58.543	0.106	−0.562	0.575	27.907	58.996	−0.09	−0.473	0.637	73.986	59.456	0.237	−1.244	0.216
second	18.859	41.901	0.06	0.45	0.654	23.252	42.348	0.074	0.549	0.584	−4.849	43.107	−0.015	−0.112	0.911
third	57.199	45.923	0.21	1.246	0.216	61.172	46.284	0.225	1.322	0.189	22.461	46.958	0.083	0.478	0.633
Academic grades															
4.0≤	−28.406	32.624	−0.077	−0.871	0.386	−28.663	32.685	−0.078	− 0.877	0.383	− 23.244	31.585	− 0.063	− 0.736	0.463
3.0–3.99	−12.027	23.923	−0.042	− 0.503	0.616	− 11.718	23.969	− 0.041	− 0.489	0.626	−6.857	23.571	− 0.024	− 0.291	0.772
CPS	3.311	9.89	0.024	0.335	0.738	2.246	10 < .001	0.016	0.225	0.823	7.509	9.872	0.055	0.761	0.449
MS	1.898	10.639	0.014	0.178	0.859	3.284	10.804	0.024	0.304	0.762	−3.038	10.528	−0.022	− 0.289	0.774
EBVNV															
yes	17.288	19.721	0.064	0.877	0.383	17.003	19.76	0.063	0.86	0.391	14.758	19.037	0.055	0.775	0.44
AC	−7.255	14.701	−0.054	−0.494	0.623	−6.416	14.766	−0.047	− 0.435	0.665	−15.33	14.527	−0.113	−1.055	0.294
BMI (kg/m^2^)	−1.77	9.278	−0.013	−0.191	0.849	−1.77	9.295	−0.013	−0.19	0.849	1.725	9.388	0.012	0.184	0.855
Knowledge	12.99	9.535	0.096	1.362	0.176	12.142	9.613	0.089	1.263	0.209	11.123	9.273	0.082	1.2	0.233
Performance confidence	−4.618	9.465	−0.035	−0.488	0.627	−5.982	9.64	−0.045	−0.621	0.536	−2.116	9.422	−0.016	−0.225	0.823
C.						−6.774	8.63	−0.05	−0.785	0.434	−0.135	9.095	−0.001	−0.015	0.988
PP × C.											25.179	10.33	0.195	2.437^*^	0.017
ln (ASF) × C.											20.637	8.287	0.154	2.490^*^	0.014
BSF × C.											5.286	9.198	0.035	0.575	0.567
LSF × C.											−9.387	7.948	−0.071	−1.181	0.24
RSF × C.											−20.306	13.815	−0.129	−1.47	0.145
	F = 11.210^***^, R^2^(_adj_ R^2^) = .656(.597)					F = 10.614^***^, R^2^(_adj_ R^2^) = .658(.596)					F = 9.676^***^, R^2^(_adj_ R^2^) = .699(.627)				

*PP* Peak pressure, *ASF* Apex sealing force, *BSF* Bottom sealing force, *LSF* Left sealing force, *RSF* Right sealing force, *CPS* Clinical practice satisfaction, *MS* Major satisfaction, *EBVNV* Experience of BVM ventilation, *AC* Acquired certificate, *C.* C length

ln (ASP) = ln (ASP+ 1)^8;, *^p < 0.05, ^**^p < 0.01, ^***^p < 0.001;Durbin-Watson = 1.928

## Discussion

Focusing on C length, this study aimed to identify the factors affecting the TV via the 4-point sealing forces of the mask in BVM ventilation, using the one-handed technique, among undergraduate paramedic students from two universities in South Korea. Regression analysis of the moderating effects of C length revealed moderation of the effects of apex sealing force and PP on TV. Thus, the greater the PP and apex sealing force, the greater the TV; similarly, the longer the C length, the greater the TV.

The average C length in this study was 7.54 cm, with a longer C length being associated with a greater TV; however, a previous study [14] reported that the EO technique, where the distance from the thumb to the index finger is 0 cm, provided more TV than the EC technique. In the novice group, BVM ventilation was more effective using the EO technique than using the thenar eminence or the EC technique [15]. Traditionally, the EC grip has been recommended for effective BVM ventilation by the AHA guidelines. The C length was measured as a variable affecting TV in this study; however, this was considerably different in previous studies [7, 8, 10, 11, 14] wherein the hand length (distance from the middle edge of the palm to the distal thumb) and width (distance from the middle fingertip to the distal skin crease at the wrist) were measured as TV-related variables. It is difficult to compare the results of these previous studies with those of the current study, as the measurement criteria for hand size differed. Nevertheless, BVM ventilation is traditionally performed by the EC grip; in addition to the other variables, C length may be a significant determinant of TV and hence, successful bagging.

The PP is the highest level of pressure applied to the lungs during inhalation. In this study, the average PP was 11.62 cmH_2_O, which was lower than that in a previous study [13]; however, this was higher than the 8.71 cmH_2_O previously reported [17]. The PP is directly proportional to TV; a low PP was associated with suboptimal TV (321.6 mL) in this study. A previous study [23] reported that a PP > 20 cmH_2_O resulted in adverse effects, such as gastric insufflation. A higher PP (above 40 cmH_2_O) is associated with higher hospital mortality [24–26]. From the clinical perspective, appropriate BVM ventilation is important; however, it is similarly important to avoid adverse effects from excessive PP.

The average range of the 4-point sealing forces was 0.03–0.69 N. There are no previous studies measuring mask sealing force in BVM ventilation; this is the first simulation study measuring the 4-point sealing forces in BVM ventilation as a variable affecting TV. We believe that the mask sealing force results provide a hypothesis for future studies, as the apex sealing force was the weakest among the 4-point sealing forces in this study. It may be that the upward pressure of the three fingers, forming the letter “E” on the inferior of the mandible to hold the mask to the face, was stronger than the downward pressure of the thumb and forefinger, forming “C” shape in the 1-handed technique, on the mask. Nevertheless, in-depth future studies are needed to confirm whether the apex sealing force is 10 times lower than the sealing force of the other three points. Previous studies [8, 11] on the effect of handgrip strength on BVM ventilation were inconsistent. The results of this study suggest more technicalities than those of previous studies. For the development of effective BVM ventilation, continuous research on sealing force in various clinical situations is needed.

The average TV (321.6 mL) in this study was comparable with that of a previous study [14]; participants in this study did not achieve adequate TV, even though they had completed the CPR theory and practical courses according to the AHA guidelines. We are not certain whether this result is related to the ability to perform the procedure or the lack of proper education; however, the evaluation of students’ appropriate performance is as important as the education, and the AHA BLS-HP course does not provide feedback regarding BVM ventilation. Although simple, BVM ventilation is an important emergency airway management technique that requires skill; therefore, ongoing education and training are necessary to enhance the effective sealing technique, creating a tighter seal between the mask and face, of paramedic students who will work in the prehospital stage.

### Limitations

First, a manikin model was used instead of humans; although the manikin model allowed us to test our study in a highly controlled and consistent environment, it is unclear whether the same results would be achieved in patients. Second, the study results may not extend to all cases of BVM ventilation, as we only selected participants through convenience sampling. Additionally, this study did not use nasal or oral airways and did not consider the patient’s facial characteristics.

## Conclusions

Based on these results, the most crucial point in the proper delivery of TV during BVM ventilation is how well the mask is closely sealed with the patient’s face without air leaking. Therefore, it is necessary to educate through programs the use of equipment with a feedback device that can provide accurate information on 4-point sealing forces. In addition, we believe that the results of this study suggest a new criterion for effective BVM ventilation in clinical practice and future research.

## Data Availability

The datasets used and analyzed during this study are available from the corresponding author on reasonable request.

## Abbreviations

AHA: American Heart Association
BVM: Bag-valve-mask
CPR: Cardiopulmonary resuscitation
PP: Peak pressure
TV: Tidal volume
